# Recent Emergence of Dengue Virus Serotype 4 in French Polynesia Results from Multiple Introductions from Other South Pacific Islands

**DOI:** 10.1371/journal.pone.0029555

**Published:** 2011-12-28

**Authors:** Van-Mai Cao-Lormeau, Claudine Roche, Maite Aubry, Anita Teissier, Stéphane Lastere, Elise Daudens, Henri-Pierre Mallet, Didier Musso, John Aaskov

**Affiliations:** 1 Laboratoire de recherche en virologie médicale, Institut Louis Malardé, Papeete, Tahiti, French Polynesia; 2 Laboratoire d'analyses de biologie médicale, Institut Louis Malardé, Papeete, Tahiti, French Polynesia; 3 Bureau de veille sanitaire, Direction de la santé, Papeete, Tahiti, French Polynesia; 4 Queensland University of Technology, Brisbane, Australia; Duke-National University of Singapore Graduate Medical School, Singapore

## Abstract

**Background:**

Infection by dengue virus (DENV) is a major public health concern in hundreds of tropical and subtropical countries. French Polynesia (FP) regularly experiences epidemics that initiate, or are consecutive to, DENV circulation in other South Pacific Island Countries (SPICs). In January 2009, after a decade of serotype 1 (DENV-1) circulation, the first cases of DENV-4 infection were reported in FP. Two months later a new epidemic emerged, occurring about 20 years after the previous circulation of DENV-4 in FP. In this study, we investigated the epidemiological and molecular characteristics of the introduction, spread and genetic microevolution of DENV-4 in FP.

**Methodology/Principal Findings:**

Epidemiological data suggested that recent transmission of DENV-4 in FP started in the Leeward Islands and this serotype quickly displaced DENV-1 throughout FP. Phylogenetic analyses of the nucleotide sequences of the envelope (E) gene of 64 DENV-4 strains collected in FP in the 1980s and in 2009–2010, and some additional strains from other SPICs showed that DENV-4 strains from the SPICs were distributed into genotypes IIa and IIb. Recent FP strains were distributed into two clusters, each comprising viruses from other but distinct SPICs, suggesting that emergence of DENV-4 in FP in 2009 resulted from multiple introductions. Otherwise, we observed that almost all strains collected in the SPICs in the 1980s exhibit an amino acid (aa) substitution V287I within domain I of the E protein, and all recent South Pacific strains exhibit a T365I substitution within domain III.

**Conclusions/Significance:**

This study confirmed the cyclic re-emergence and displacement of DENV serotypes in FP. Otherwise, our results showed that specific aa substitutions on the E protein were present on all DENV-4 strains circulating in SPICs. These substitutions probably acquired and subsequently conserved could reflect a founder effect to be associated with epidemiological, geographical, eco-biological and social specificities in SPICs.

## Introduction

Almost all tropical and subtropical regions of the world are concerned by the risk of dengue. Every year, dengue virus (DENV) causes more than 50 million infections, 500 000 hospitalizations and 12 500 deaths, mostly children [1]. Currently there is no vaccine or effective anti-viral therapy and vector control measures regularly fail to prevent the emergence of dengue epidemics. DENV is a single-stranded, positive-sense RNA virus belonging to the genus *Flavivirus*, family *Flaviviridae*, transmitted by *Aedes* mosquitoes, principally *Aedes (Stegomyia) aegypti* but also *Aedes albopictus* and some endemic vectors, like *Aedes polynesiensis* in the Polynesian triangle [2]. Infection with dengue virus may result in a wide spectrum of clinical manifestations accounting for the classification of the disease as dengue (fever, rash, headache, myalgia, arthralgia, nausea, vomiting) with or without warning signs (abdominal pain, persistent vomiting, clinical fluid accumulation, mucosal bleed) or severe dengue (characterized by plasma leakage) with or without hemorrhage [3]. There are four distinct serotypes of dengue virus (DENV-1 to -4) and infection with one serotype provides no long-term cross-protective immunity against the three others. Based on the sequence of the envelope gene (E), each serotype may be divided into distinct phylogenetic clusters or genotypes [4]. Both epidemiological observations and *in vitro* studies suggest that some genotypes may have different epidemic potential [5]–[7].

In the South Pacific, the earliest dengue epidemics may have occurred during the nineteenth century [8]. However, dengue only became a significant health problem during World War II, because of the spread of the main dengue vector, *Ae. aegypti*. The first documented outbreak of DENV in the South Pacific occurred in 1943–1944 and was due to serotype 1. When dengue reappeared 20 years later in French Polynesia (FP) it was due to DENV-3 [9]–[10]. The first cases of severe dengue in the Pacific were reported in the 1970s [11]–[13]. Beginning in 2000, outbreaks of DENV in South Pacific Island Countries (SPICs) have been due to DENV-1 [14]–[19] but in 2008, DENV-4 circulation was detected [20]–[22] for the first time since the 1980's [23], [24]. In January 2009, about 20 years after the last reported case, DENV-4 was detected in two members from the same family returning to FP from New Caledonia (NC) where this serotype had recently been detected. Two months later FP experienced a large dengue outbreak.

Here, we report the epidemiological and molecular characteristics of the introduction, spread and genetic microevolution of DENV-4 in FP.

## Materials and Methods

### Epidemiological and biological data

Laboratory diagnosis of dengue was performed on sera from dengue suspected patients using ELISA tests for the detection of either DENV NS1 (Platelia™ Dengue NS1 Ag, Bio-Rad Laboratories, Marnes-la-Coquette, France) or anti-DENV IgM (Dengue IgM Capture ELISA, Panbio Diagnostics, Brisbane, Australia). A DENV serotype-specific multiplex real-time RT-PCR using primers and probes designed by Johnson *et al.* (2005) [25] was performed on sera containing DENV NS1 or from patients who have travelled recently to areas where dengue is known to occur. Data from January 2009 to December 2010 were recorded by the Health Department (Bureau de Veille Sanitaire, Tahiti, French Polynesia) based on reports transmitted by Institut Louis Malardé (ILM, Tahiti, French Polynesia) and the principal hospital in Tahiti (Centre Hospitalier de la Polynésie Française).

### Viruses

The details of the viruses employed are shown in Table 1. Briefly, DENV-4 strains collected in FP in the 1980s were obtained from the DENV collection of ILM. At the time they were collected, these strains were amplified by one passage on C6/36 *Ae. albopictus* cells and cell supernatants were then preserved at −80°C. DENV-4 strains recently collected in FP were obtained from sera positive by DENV-4 real-time RT-PCR performed by the clinical laboratory from ILM. One of these sera was recovered from a patient recently returned from NC [PF-NC09/160109-136]. Two additional DENV-4 strains collected in NC and Wallis & Futuna [NC09/060209-1528, WF09/010409-0001] were kindly provided by Dr M Dupont-Rouzeyrol, Institut Pasteur de Nouvelle Calédonie, and Dr JF Yvon, clinical laboratory from Sia Hospital, Wallis. Five additional strains collected in the SPICs in 2008–2009 [Kiribati KI08/266, Tonga TO08/14, Fiji FJ08/3953, Vanuatu VU09/4214 and Western Samoa WS08/73] were sequenced as previously described [22]. Additional E gene sequences of 73 strains were retrieved from GenBank.

**Table 1:** Characteristics of the 64 DENV-4 strains used for E-gene sequence analysis.

ID	Country	Archipelago	Island	District	Year	GenBank
NC09/060209-1528	NC				2009	JN832498
WF09/010409-0001	WF				2009	JN832499
PF-NC09/160109-136	PF (imp. from NC)		Tahiti	Mahina	2009	JN832507
PF82/5918	PF	Windward Islands	Tahiti		1982	JN832500
PF83/6074			Tahiti		1983	JN832501
PF84/131-188			Tahiti		1984	JN832502
PF85/323-103			Tahiti		1985	JN832503
PF86/50-70			Tahiti		1986	JN832504
PF87/19-7			Tahiti		1987	JN832505
PF09/060309-96			Tahiti	Papeari	2009	JN832514
PF09/130309-109			Tahiti	Punaauia	2009	JN832519
PF09/160309-24			Tahiti	Mahina	2009	JN832520
PF09/230309-126			Tahiti	Punaauia	2009	JN832521
PF09/310309-162			Tahiti	Pirae	2009	JN832524
PF09/270409-182			Tahiti	Faaa	2009	JN832530
PF09/150609-91*			Tahiti	Punaauia	2009	JN832536
PF09/220709-54*			Tahiti	Punaauia	2009	JN832541
PF09/220909-50			Tahiti	Papeete	2009	JN832545
PF09/141009-111			Tahiti	Papeete	2009	JN832546
PF09/041109-131			Tahiti	Papara	2009	JN832547
PF09/041209-32			Tahiti	Arue	2009	JN832548
PF10/260110-67			Tahiti	Punaauia	2010	JN832551
PF10/050210-117			Tahiti	Punaauia	2010	JN832552
PF10/120310-31			Tahiti	Papeete	2010	JN832554
PF10/010410-215			Tahiti	Mahina	2010	JN832555
PF10/060410-11			Tahiti	Papeete	2010	JN832556
PF10/300410-48			Tahiti	Punaauia	2010	JN832557
PF10/170510-92			Tahiti		2010	JN832558
PF10/170510-191			Tahiti	Punaauia	2010	JN832559
PF10/170510-192			Tahiti	Punaauia	2010	JN832560
PF10/150610-28			Tahiti	Papara	2010	JN832561
PF09/020309-54			Moorea		2009	JN832512
PF09/190809-58			Moorea		2009	JN832543
PF10/190110-98			Moorea		2010	JN832550
PF10/010310-32			Moorea		2010	JN832553
PF09/090309-122		Leeward Islands	Raiatea		2009	JN832516
PF09/080409-93			Raiatea		2009	JN832526
PF09/040509-242			Raiatea		2009	JN832531
PF09/120609-125			Raiatea		2009	JN832535
PF09/270709-74			Raiatea		2009	JN832542
PF09/230209-116			Bora Bora		2009	JN832509
PF09/100309-172			Bora Bora		2009	JN832517
PF09/100309-208			Bora Bora		2009	JN832518
PF09/190509-238			Bora Bora		2009	JN832533
PF09/200809-131			Bora Bora		2009	JN832544
PF09/290109-69			Taha'a		2009	JN832508
PF09/270209-213			Taha'a		2009	JN832511
PF09/030309-39			Taha'a		2009	JN832513
PF09/060309-198			Taha'a		2009	JN832515
PF09/210409-134			Maupiti		2009	JN832527
PF09/190509-37			Maupiti		2009	JN832532
PF09/160609-119			Maupiti		2009	JN832537
PF88/130-74		Marquesas archipelago			1988	JN832506
PF09/240209-38			Ua Pou		2009	JN832510
PF09/270309-27			Ua Pou		2009	JN832522
PF09/270309-31			Ua Pou		2009	JN832523
PF09/081209-283			UaPou		2009	JN832549
PF09/290509-25			Nuku Hiva		2009	JN832534
PF09/070409-227		Australes archipelago	Rimatara		2009	JN832525
PF09/230409-06			Rurutu		2009	JN832528
PF09/250609-14			Rurutu		2009	JN832538
PF09/240409-72		Tuamotu archipelago	Hao		2009	JN832529
PF09/210709-11			Hao		2009	JN832540
PF09/080709-23			Manihi		2009	JN832539

*patient with severe dengue.

### Ethics statement

All dengue virus strains were obtained from sera initially sampled for dengue diagnostic and surveillance purposes (under medical prescription by a physician), and archived at ILM. The only information provided to the research laboratory was the sample laboratory ID number, the date of collection and the district/island of collection (no personal information on the patient was provided). The use of biological samples and the collection of information were performed in accordance with the French regulations.

### Nucleotide sequencing

Sera or whole blood on filter paper cards (LDA^22^, France) were used as a source of DENV. RNA was extracted from sera using the QIAamp® Viral RNA Mini Kit (Qiagen, Germany) or the Easymag extraction system (Biomérieux, France) according to manufacturers' instructions. For whole blood, five filter paper spots per patient were incubated for 20 min at room temperature in lysis buffer from the QIAamp® Viral RNA Mini Kit. After centrifugation for 10 min at 8000 rpm, the extraction process was pursued using the supernatant, according to the manufacturer's instructions. Amplification of the E gene was then performed using the OneStep RT-PCR® Kit (Qiagen, Germany) and two manually designed oligonucleotides primer pairs [D4E/777F (5′- *GCT TGG AAA CAT GCT CAG AG*-3′) and D4E/1766R (5′- *ACA TGT GGT TTC CAT CAC CG-*3′); D4E/1639F (5′-*TGG TGA CAT TCA AGG TTC CTC*-3′) and D4E/2509R (5′-*ACT GTT CTG TCC AAG TGT GC*-3′)] in order to produce two overlapping fragments covering the complete E gene. For two sequences [PF09/270309-27, PF09/270309-31], a second round of PCR was required to obtain sufficient DNA. The PCR products were purified using the QIAquick PCR Purification Kit (Qiagen, Germany), the subsequent sequencing reactions performed with the ABI PRISM® BigDye™ Terminator Cycle Sequencing Ready Reaction Kit (Applied Biosystems, USA). and the product analyzed on the ABI Prism 310 automated sequencer (Perkin-Elmer, Applied Biosystems). Overlapping nucleotide sequences were assembled with AutoAssembler software (Perkin Elmer). Nucleotide sequences were aligned with the multiple sequence alignment software ClustalW integrated in MEGA version 5 [26]. Phylogenetic analysis was performed with MEGA5 using the Maximum likelihood based on the Kimura 2-parameter method [27]. The robustness of the original tree was tested with 1000 bootstrap replications [28]. Sequences available on GenBank and corresponding to strains belonging to all major branches of previously published DENV-4 phylogenies were included in the analysis and selected to maximize the representation of genotypes, countries and years of isolation. Those that were very closely related to one another in the data set were removed from the analysis. Mean distances between and within groups were calculated for all sequences (MEGA5).

## Results

### Epidemiology

**Figure 1:**
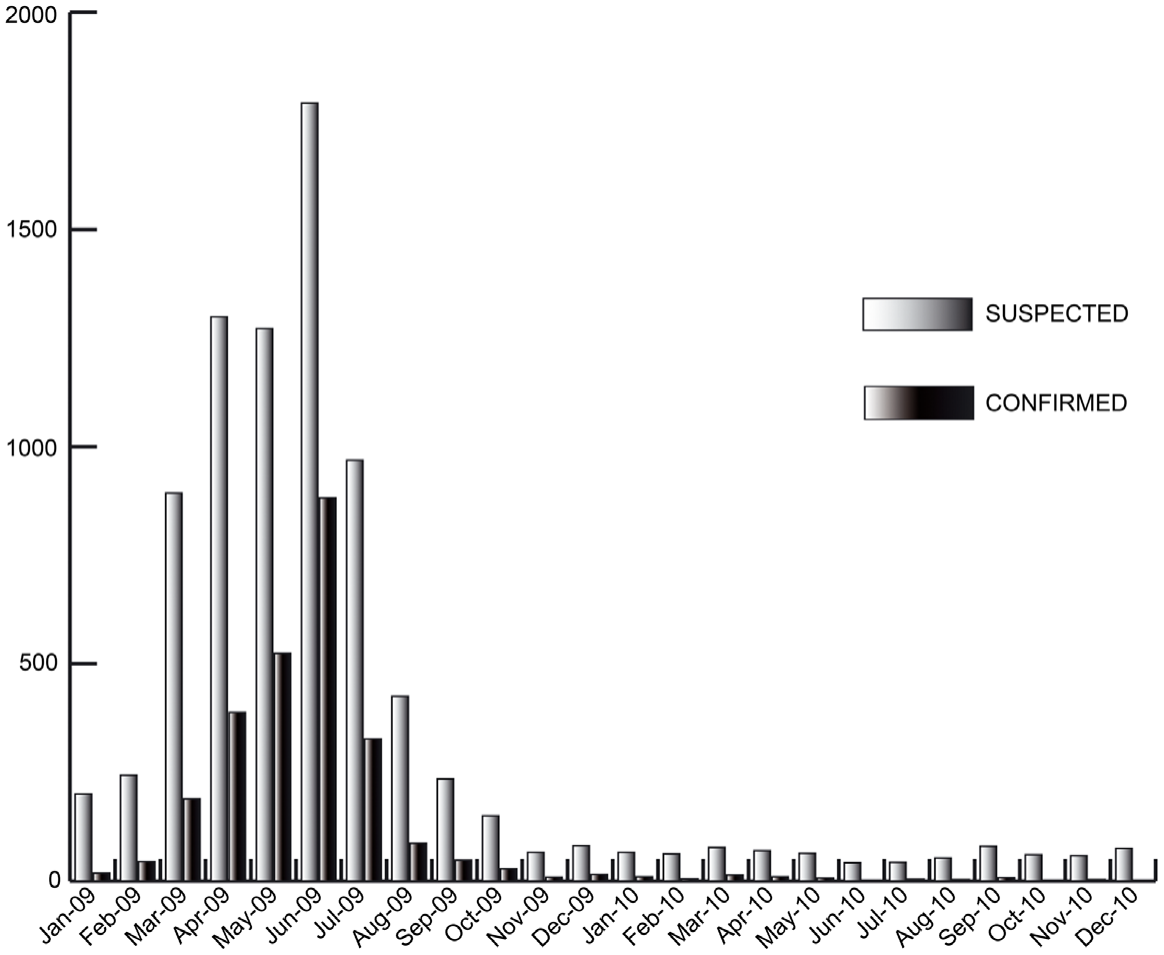
Monthly recorded dengue suspected and confirmed cases from January 2009 to December 2010.

Two patients with DENV-4 infections were detected in January 2009 in Tahiti, Society Islands. The patients were members from the same family recently returned from NC where a DENV-4 outbreak had recently commenced concomitantly with DENV-1 transmission. These were the first cases of infection with DENV-4 detected in FP for more than 20 years. The French Polynesia Public Health and Hygiene Department immediately applied vector control measures by spraying insecticide against adult mosquitoes and destroying breeding sites around the home of these patients. The insecticide treatment was repeated 10 days later. No new DENV-4 cases were detected in Tahiti in the following weeks. However, at the end of February, DENV-4 was detected in three patients living in Taha'a, five in Bora-Bora, one in Tahiti (Society Islands) and one in Ua Pou (Marquesas Islands). None of these patients had traveled out from FP during the two previous weeks. An additional patient from Taha'a with an onset of symptoms at the end of January was found subsequently, by RT-PCR, to also have had a DENV-4 infection. From January to March 2009, the number of patients with DENV-4 infections increased dramatically and DENV-4 had displaced DENV-1 3 months later (Table 2). From January 2009 to December 2010, ILM reported 8375 presumptive cases of dengue to the Health Department, of which 2629 were confirmed subsequently by serological or virological testing. One hundred of these were due to DENV-1 and 763 to DENV-4 infections (Figure 1). During this period, 87 hospitalizations were reported of which three were dengue severe cases.

**Table 2:** Spatial and temporal distribution of confirmed cases of DENV-1 and DENV-4 infections between January and December 2009.

		Society archipelago		Marquesas archipelago	Austral archipelago	Tuamotu archipelago	Gambier archipelago
	Total	Windward Islands	Leeward Islands				
**January**							
DENV-1	11	Tahiti 11					
DENV-4	3	Tahiti 2	Tahaa 1				
**February**							
DENV-1	29	Tahiti 29					
DENV-4	10	Tahiti 1	Tahaa 3	Ua Pou 1			
			Bora Bora 5				
**March**							
DENV-1	39	Tahiti 37					
		Moorea 2					
DENV-4	132	Tahiti 41	Tahaa 42	Ua Pou 3			
		Moorea 3	Bora Bora 33				
			Raiatea 10				
**April**							
DENV-1	20	Tahiti 17	Raiatea 1				
		Moorea 2					
DENV-4	262	Tahiti 130	Tahaa 14	Ua Pou 8	Rimatara 1	Hao 2	
		Moorea 25	Bora Bora 55	Nuku-Hiva 1	Rurutu 2		
			Raiatea 21	Fatu-Hiva 1			
			Maupiti 2				
**May**							
DENV-1	2	Tahiti 2					
DENV-4	126	Tahiti 80	Bora Bora 9	Ua Pou 4	Tubuai 1		
		Moorea 10	Raiatea 9	Nuku-Hiva 6			
			Maupiti 4	Ua Huka 1			
			Huahine 2				
**June**							
DENV-1	1	Tahiti 1					
DENV-4	165	Tahiti 112	Bora Bora 4	Nuku-Hiva 1	Rurutu 6	Hao 4	
		Moorea 13	Raiatea 15		Raivavae 2	Fakarava 3	
			Maupiti 2			Manihi 1	
			Huahine 1			Rangiroa 1	
**July**							
DENV-4	35	Tahiti 23	Raiatea 1	Fatu-Hiva 1		Hao 1	
		Moorea 4	Huahine 1			Manihi 4	
**August**							
DENV-4	7	Tahiti 3	Bora Bora 1			Hao 1	
		Moorea 2					
**September**							
DENV-4	9	Tahiti 5		Nuku-Hiva 2			Mangareva 1
				Hiva-Oa 1			
**October**							
DENV-4	5	Tahiti 4				Rangiroa 1	
**November**							
DENV-4	2	Tahiti 2					
**December**							
DENV-4	7	Tahiti 6		Ua Pou 1			

### Molecular epidemiology of DENV-4 in FP

**Figure 2:**
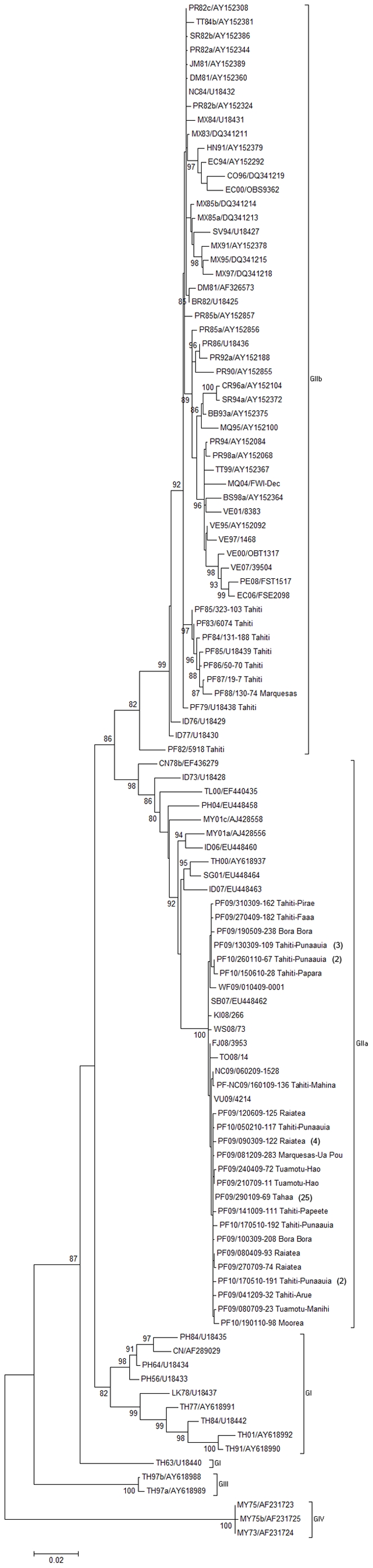
Evolutionary relationships of DENV-4 E gene sequences. ML original tree derived from 110 DENV-4 E gene sequences. The percentage of replicate trees in which the associated taxa clustered together in the bootstrap test (1000 replicates) is shown for values over 80. The number of strains with identical E gene sequence is indicated in parenthesis (these additional strains could have been collected in a different district or island and at a different date than the strain that appears in the tree).

**Figure 3:**
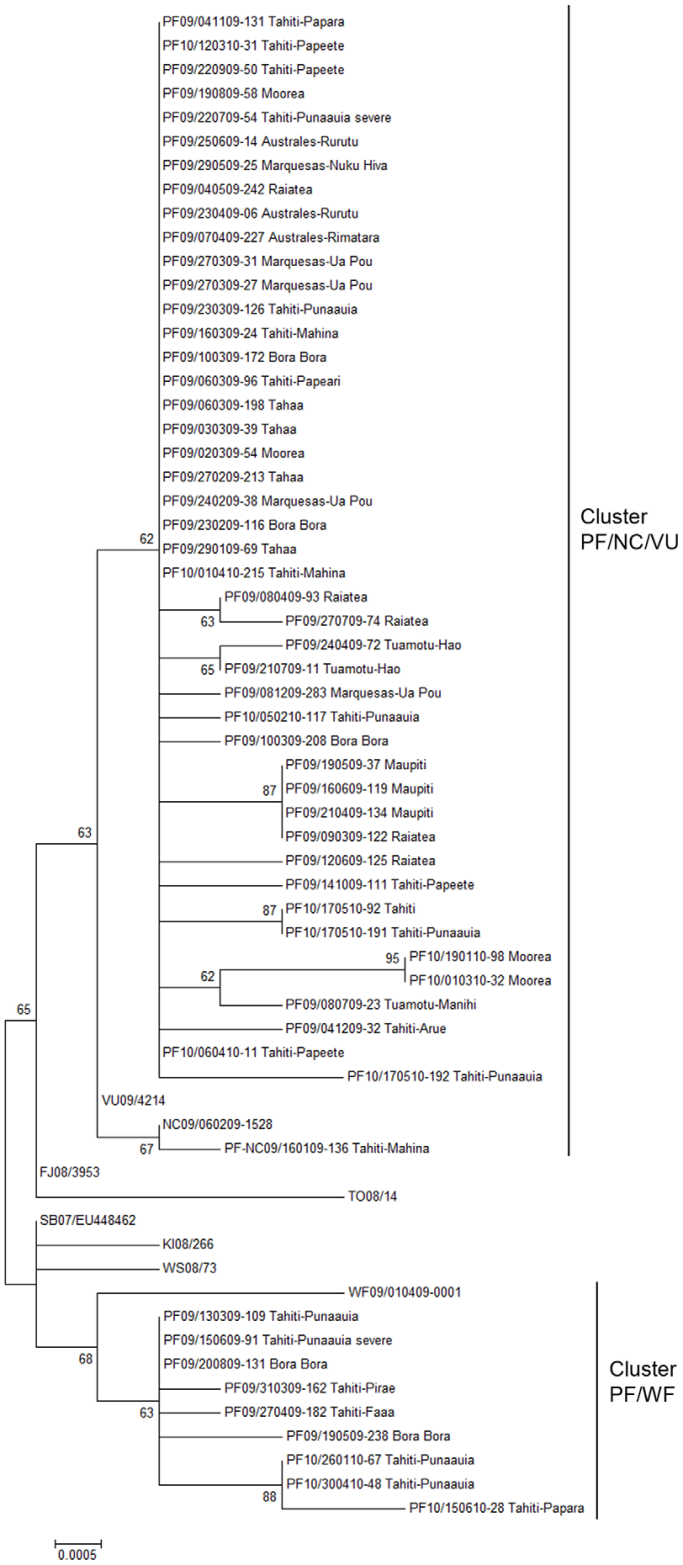
Evolutionary relationships of DENV-4 Oceania group within clade IIa. ML original tree derived from 63 DENV-4 E gene sequences. The percentage of replicate trees in which the associated taxa clustered together in the bootstrap test (1000 replicates) is shown next to the branches.

The 64 E gene sequences generated in the present study were combined with 5 E gene sequences from DENV-4 strains collected in the South Pacific between 2008 and 2009 and a data set of 73 E gene sequences available on GenBank representing the global genetic variability of DENV-4 (Figure 2). The phylogenetic tree shows four distinct clusters named genotype I, II, III and IV. These four clusters have been previously described as genotype I or Southeast Asia, II or Indonesia, III, and IV or Sylvatic or Malaysia [6], [29]–[31]. All sequences of DENV-4 collected in the South Pacific belonged to genotype II. The average distances between genotype II and the three others varied from 0.073 to 0.156, with standard errors (SE) of 0.005 and 0.011 respectively. Within genotype II, viruses clustered into two distinct clades previously defined as IIa and IIb [32]. The average distance between these two clades is 0.063 with a SE of 0.007. The Clade IIb consisted of strains isolated from 1979 to 2008 in the South Pacific, the Caribbean, South and Central America, along with two strains isolated in Indonesia in 1976 and 1977. Within clade IIb, viruses collected in FP from 1983 to 1988 formed a distinct group supported by a bootstrap value of 99% which we have called the PF80s group. Clade IIa was composed, almost exclusively, of viruses collected in the South Pacific and Southeast Asia since 2000. Within clade IIa, all DENV-4 strains collected in the South Pacific from 2007 to 2010 formed a distinct group supported by a bootstrap value of 100% and which we have named the Oceania group. Within the Oceania group, the strains collected in FP were distributed into two clusters (Figure 3). The first one, PF/NC/VU, comprised most (83%) of the FP strains and was close to the strains from NC and Vanuatu. The second cluster, PF/WF, included nine FP strains and was closer to the strain from Wallis & Futuna. Within each cluster, several FP strains shared identical E gene consensus sequences.

Based on the Solomon Island [SB07/EU448462] E gene sequence, 53 sites exhibited a nucleotide (nt) substitution of which 18 were present on more than one strain but didn't lead to an amino acid (aa) substitution (Table 3). The analysis of the 142 E gene sequences included in this study revealed 79 aa mutated sites, of which one aa substitution (T365I) was specifically encountered within the Oceania group and another (V287I) was only found in the PF80s group (Table 4).

**Table 3:** Significant nucleotide substitutions on the E gene (1485 bp) within the DENV-4 clade IIa Oceania group.

		nt positions																	
Cluster	Strains	15	63	213	273	375	498	549	606	657	738	885	957	978	1146	1365	1386	1446	1465
**none**	SB07/EU448462	G	C	A	T	A	C	C	A	T	G	G	A	T	T	T	A	T	C
	KI08/266	-	-	-	-	-	-	-	-	-	-	-	-	-	-	-	-	-	-
	WS08/73	-	-	-	-	-	-	-	-	-	-	-	-	-	-	-	-	-	-
**PF/WF**	WF09/010409-0001	-	-	-	-	T	-	-	-	-	-	-	-	-	-	-	-	-	-
	Punaauia 09 Group (3 strains)	-	-	-	-	T	-	-	-	-	-	-	-	-	-	-	-	-	T
	PF09/310309-162 Tahiti-Pirae	-	-	-	-	T	-	-	-	-	-	-	-	-	-	-	-	-	T
	PF09/270409-182 Tahiti-Faaa	-	-	-	-	T	-	-	-	-	-	-	-	-	-	-	-	-	T
	PF09/190509-238 Bora Bora	-	-	-	-	T	-	-	-	-	-	-	-	-	-	-	-	-	T
	Punaauia 10a Group (2 strains)	-	-	-	-	T	-	-	-	C	-	-	-	-	-	C	-	-	T
	PF10/150610-28 Tahiti-Papara	-	-	-	-	T	-	-	-	C	-	-	-	-	-	C	-	-	T
**none**	FJ08/3953	-	-	-	-	-	-	-	-	-	-	-	-	-	-	-	G	-	-
	TO08/14	-	-	-	-	-	-	-	-	-	-	-	-	-	-	-	G	-	-
**PF/NC/VU**	VU09/4214	-	-	-	-	-	-	-	-	-	A	-	-	-	-	-	G	-	-
	PF-NC09/160109-136 Tahiti-Mahina	-	-	-	-	-	-	T	-	-	A	-	-	-	-	-	G	-	-
	NC09/060209-1528	-	-	-	-	-	-	T	-	-	A	-	-	-	A	-	G	-	-
	PF09/10 Group (25 strains)	-	-	-	-	-	-	-	-	-	A	-	-	-	A	-	G	-	-
	PF09/100309-208 Bora Bora	-	-	-	-	-	-	-	-	-	A	-	-	-	A	-	G	-	-
	PF09/120609-125 Raiatea	-	-	-	-	-	-	-	-	-	A	-	-	-	A	-	G	-	-
	PF09/141009-111 Tahiti-Papeete	-	-	-	-	-	-	-	-	-	A	-	-	-	A	-	G	-	-
	PF09/041209-32 Tahiti-Arue	-	-	-	-	-	-	-	-	-	A	-	-	-	A	-	G	-	-
	PF09/081209-283 Marquesas-Ua Pou	-	-	-	-	-	-	-	-	-	A	-	-	-	A	-	G	-	-
	PF10/050210-117 Tahiti-Punaauia	-	-	-	-	-	-	-	-	-	A	-	-	-	A	-	G	-	-
	PF10/170510-192 Tahiti-Punaauia	-	-	-	-	-	-	-	-	-	A	-	-	-	A	-	G	-	-
	Maupiti 09 Group (4 strains)	A	-	-	C	-	-	-	-	-	A	-	-	-	A	-	G	-	-
	PF09/080409-93 Raiatea	-	-	-	-	-	-	-	-	-	A	-	G	-	A	-	G	-	-
	PF09/270709-74 Raiatea	-	-	-	-	-	-	-	-	-	A	-	G	-	A	-	G	-	-
	PF09/240409-72 Tuamotu-Hao	-	-	-	-	-	T	-	-	-	A	-	-	-	A	-	G	-	-
	PF09/210709-11 Tuamotu-Hao	-	-	-	-	-	T	-	-	-	A	-	-	-	A	-	G	-	-
	PF09/080709-23 Tuamotu-Manihi	-	T	-	-	-	-	-	-	-	A	-	-	-	A	-	G	-	-
	Moorea 10 Group (2 strains)	-	T	G	-	-	-	-	-	-	A	A	-	-	A	-	G	C	-
	Punaauia 10b Group (2 strains)	-	-	-	-	-	-	-	G	-	A	-	-	C	A	-	G	-	-

- Identical to Solomon.

**Table 4:** Relevant amino acid substitutions on the E protein based on the consensus sequence of 142 DENV-4 strains.

Clade	Group	Substitutions	Comments
**IIa**	**Oceania**	M/I34T	**TO08/14**
		I/A46T*	**Clade IIa** (except WF09/010409-1); Clade IIb (except PF82/5918)
		I/T46A	**WF09/010409-1**
		L82P	**TO08/14**
		S120L	**Clade IIa** (except MY01a)
		S156P	**TO08/14**
		V160M	**PF10/170510-192**
		V173I	**PF09/310309-162**
		N276S	**PF09/141009-111**
		K323Q	**PF09/190509-238**
		V335I	**Oceania group**, MY01a (Clade IIa); TH63 (Genotype I); Genotype IV
		T365I*	**Oceania group**
		I380V	**KI08/266**
		D/E384N*	**PF10/050210-117**, MY01a (Clade IIa); LK78 (Genotype I); Clade IIb
**IIb**	**PF80s**	I/A46T*	**Clade IIb** (except PF82/5918); Clade IIa (except WF09/010409-1)
		V/A141I	**PF85/U18439**
		D/S154G	**PF88/130-74**
		T155I	**PF82/5918**; ID73, CN78b (Clade IIa); PH56, TH63 (Genotype I)
		K/E202R	**PF79**; TH63, LK78 (Genotype I)
		T221A	**PF82/5918**; CN, PH56, PH64, PH84 (Genotype I)
		A/T222V	**PF87/19-7**
		S/L/A227T	**PF80s group** (except PF79/U18438**,** PF82/5918, PF85/323-103)
		V287I	**PF80s group** (except PF79/U18438)
		I351V	**PF80s group** (except PF79/U18438); most strains from Genotype IIb; CN, PH84 (Genotype I)
		P356L	**PF88/130-74**
		F357L	**Clade IIb**; ID73 (Genotype IIa); PH56, PH64, PH84, TH63, TH84 (Genotype I)
		D/E384N*	**Clade IIb**; PF10/050210-117, MY01(Clade IIa); LK78 (Genotype I)

*non conservative aa substitution; strains of interest are in bold characters.

## Discussion

The recent emergence of DENV-4 in the SPICs occurred after a decade of active DENV-1 circulation and about 20 years after the previous circulation of DENV-4 in the region [23], [24]. This observation is consistent with previous epidemiological data suggesting that, in the region and particularly in FP, sustained transmission of a predominant serotype/genotype occurs following a 20 to 25 years periodic cycle [33], [34]. DENV-1 was still circulating in FP when DENV-4 started to be transmitted locally, but from July 2009, by performing DENV serotype-specific RT-PCR on all sera previously tested positive using NS1 test, we only found DENV-4 (Table 2). Up to November 2011, the only DENV-1 case reported was a traveler just coming back from San-Martin, Caribbean (data not shown). Previous studies already demonstrated that the sensitivity of the Platelia™ Dengue NS1 Ag test depends on the infecting DENV serotype, however the sensitivity of the test was found to be at least identical or higher for DENV-1 compared to DENV-4 [35], [36]. Our results suggest that DENV-4 displaced DENV-1. Consistently, the displacement of the DENV serotype previously circulating by the newly introduced serotype is a characteristic that has been constantly observed in FP over time [33], [34].Since the second semester of 2008, at the time the transmission of DENV-4 in the South Pacific region was dramatically increasing, dengue surveillance was reinforced in FP. However, despite an active tracking, health authorities did not reach to prevent and control the introduction of DENV-4 in FP. Beside the two DENV-4 cases imported from NC to Tahiti in mid-January, additional introduction events that remained undetected might have occurred, particularly in the Leeward islands (Society Islands) where the firsts cases due to local transmission were reported (Table 2). Additional DENV-4 cases were then progressively reported in other FP archipelagos, the spread of the virus being supported by the high frequency of inter-island travel, mostly by plane. Reminding that the DENV-1 epidemic in 2001 also started in the Leeward Islands [23], this place should be considered as a main entry point for new emergent DENV serotypes. Consistently, the Leeward Islands, notably Bora Bora, are major touristic stations in FP.

In order to complement the epidemiological data and get a better understanding of the events that might have led to the introduction and spread of DENV-4 in FP, we conducted a phylogenetic analysis on the complete E gene of DENV-4 strains collected recently and in the 1980s in FP. We also included in the study some strains collected in other SPICs in 2008–2009 and additional sequences available on GenBank (Figure 2). The resulting phylogenetic tree displays the four distinct genotypes already described [4], [29]–[31]. Furthermore, it shows that DENV-4 strains collected in FP, both in the 1980s and in 2009–2010, belong to genotype II and are distributed in clades IIa and IIb [4], [32]. These two clades are distant from more than 6% of genetic divergence and could be therefore reasonably classified in two distinct genotypes [4]. Clearly, these clades evolved separately from a common ancestor originating from South-East Asia. Interestingly, only clade IIa continued to circulate beyond the year 2000 in Southeast Asia and in the Pacific region. In contrast strains belonging to clade IIb were isolated in Central and South America and in the Caribbean at least until 2008 [37], [38]. Indeed, DENV-4 genotype IIb is still predominating in the Americas. The replacement of genotype IIb by the genotypes predominating in Southeast Asia (I, IIa, III, IV) did not occur; although introductions of genotype I were reported in Brazil in 2007 [39]. This contrasts with the situation observed for DENV-2, where the Southeast Asian genotype totally replaced the American genotype previously circulating [40], [41].

Our results corroborate previous studies showing that both in the 1980s and in the early year 2000, DENV-4 had been introduced into the region from Southeast Asia [22], [24], [30]. In addition, we found that all FP strains along with other DENV-4 strains recently collected in the SPICs form a distinct group rooted by the Solomon Island strain [SB07/EU448462], we called Oceania group (Figure 3). Within the Oceania group, the FP strains are distributed into two distinct clusters: PF/NC/VU that also comprises strains collected in NC and Vanuatu, and PF/WF that includes the strain from Wallis & Futuna. This observation suggests that the recent emergence of DENV-4 in FP results from multiple introductions of strains circulating in different SPICs. Both clusters circulated from March 2009 to June 2010. However, PF/NC/VU is widely distributed in the different FP archipelagos whereas PF/WF only comprises strains collected in the Society archipelago. Interestingly, the early dissemination of the virus started in the Leeward Islands within PF/NC/VU and in Tahiti within PF/WF. This observation suggests that in addition to be a main entry point for emergent DENV serotypes, the Leeward Islands should also be considered as an efficient starting point for active dissemination of the virus throughout all FP. The analysis of the nt substitutions within the two clusters compared to the Solomon Island strain [SB07/EU448462] reveals two nt substitutions specific to PF/NC/VU and another substitution specific to PF/WF (Table 3). Moreover, within each cluster, all FP strains share one additional nt substitution. Particularly, all FP strains in cluster PF/WF exhibit a nt substitution (C1465T) in the genomic E/NS1 junction region which is propitious to the study of evolutionary characteristics [4]. All of these nt substitutions are synonymous, showing that no major mutational event occurred on the E gene from the first emergence of DENV-4 genotype IIa in the Solomon Islands in 2007 to the current circulation in FP. Interestingly, some specific missense mutations resulting in aa substitutions occurred on the E gene of the strains collected in Kiribati and Tonga (Table 4).

A more global analysis of the aa changes on the E protein based on the consensus aa sequence of 142 DENV-4 strains reveals mutations that are specific to strains collected in the South Pacific. Within genotype IIb, all the strains composing the PF80s group, except [PF79/U18438], exhibited the same substitution V287I within the domain I of the E gene [42]. Within genotype IIa, all the strains belonging to the Oceania group show two substitutions (V335I and T365I) already described [22], [42]. These mutations occurred within the domain III that mediates DENV binding to cellular receptors and has been proved to be highly immunogenic [42], [43]. Our results show that in the 1980s like in the recent emergence of DENV-4 in the region, specific aa substitutions might have been acquired and subsequently conserved on all strains circulating in the SPICs during periods ranging from 3 (2007–2010) to 5 (1983–1988) years. In a previous study, investigating the microevolution of DENV-1 during 6 years of sustained circulation in FP, two specific aa substitutions on the E protein, that appeared during the endemic transmission period and were rapidly stabilized, were suggestive of an adaptation to the mosquito vector [18]. In the present study, the aa substitutions on the E protein found on all DENV-4 strains collected in the SPICs could reflect a founder effect, to be associated with either one or several factors characterizing the “South Pacific Islands context”: insularity, climate, presence of endemic vector species, small human population sizes and low population flows.
